# Prevalence of Musculoskeletal Conditions in Heart Patients Undergoing Cardiovascular Rehabilitation Programs: A Systematic Review and Meta-Analysis

**DOI:** 10.7759/cureus.94542

**Published:** 2025-10-14

**Authors:** Alessandra Straioto Salomão, Fernando Soares Piquione, Crystian Bitencourt Soares de Oliveira, Ana Clara Campagnolo Gonçalves Toledo

**Affiliations:** 1 Health Sciences, Universidade do Oeste Paulista (UNOESTE), Presidente Prudente, BRA

**Keywords:** cardiac rehabilitation, cardiovascular diseases, exercise training, functional limitations, meta-analysis, musculoskeletal conditions, prevalence, systematic review

## Abstract

Exercise-based cardiovascular rehabilitation is an essential strategy for managing cardiovascular diseases. However, some factors, such as musculoskeletal conditions, may limit exercise capacity and adherence. Although musculoskeletal conditions can lead to activity limitations and influence the strategies used in cardiovascular rehabilitation programs, their management and evaluation are often under-recognized. Knowledge of these conditions is important for interprofessional providers to adapt therapeutic care for this population. This study aimed to verify, through a systematic review and meta-analysis, the prevalence of musculoskeletal conditions in patients enrolled in cardiovascular rehabilitation programs. The search strategy was updated and last executed in May 2024 in the following electronic databases: MEDLINE, EMBASE, CINAHL, Web of Science, and SCOPUS. Observational studies that showed the prevalence of musculoskeletal conditions in patients with heart disease undergoing cardiovascular rehabilitation programs were eligible. To assess the risk of bias in the studies, we used the Newcastle-Ottawa Scale, and to check the overall quality of the evidence, we used the GRADE system. A total of 2,638 studies were screened, of which eight met the inclusion criteria and were rated as having moderate to high methodological quality. The prevalence of musculoskeletal conditions in patients enrolled in a cardiac rehabilitation program was 57.5%, CI 95%: 40.1-73.3; I²: 98%. Common musculoskeletal conditions were arthritis with a prevalence of 49.0%, CI 95%: 35.7-62.4; I²: 98%, musculoskeletal pain with a prevalence of 40.0%, CI 95%: 23.0-60.0; I²: 98%, and osteoporosis with a prevalence of 10.3%, CI 95%: 8.3-12.7; I²: 1%. In the studies included in this review, we found a high prevalence of musculoskeletal conditions. Future studies will be necessary to scrutinize and analyze whether the type, intensity, frequency, and duration of physical exercises proposed in cardiovascular rehabilitation programs can improve or worsen musculoskeletal conditions. These findings highlight the need to integrate musculoskeletal assessment and management into cardiac rehabilitation practice.

## Introduction and background

Cardiovascular diseases are the leading cause of death worldwide [1]. According to the World Health Organization [2], it is estimated that 17.9 million people died from cardiovascular disease in 2019, accounting for 32% of all global deaths. One of the treatment strategies for cardiovascular diseases is cardiovascular rehabilitation [3]. This multidisciplinary approach enables the application of psychosocial and physical interventions that improve health and quality of life while reducing cardiovascular and global morbidity and mortality in individuals with cardiovascular diseases.

Exercise prescriptions in cardiovascular rehabilitation must be prescribed individually for each patient according to the clinical condition, risk stratification, and objectives. This prescription should include the type of exercise, intensity, duration, and frequency [4]. However, some factors can restrict the execution of exercises proposed in cardiovascular rehabilitation programs, including musculoskeletal conditions [5].

Musculoskeletal conditions [6] are characterized by pain, impaired physical function, and limitations in activities of daily living and can lead to loss of mobility, disability, and dependence. Marzolini et al. [7] showed in their study that half of all participants who enter the cardiovascular rehabilitation program have musculoskeletal conditions. This structure may be related to the profile of patients entering cardiovascular rehabilitation programs [8], who are generally elderly, sedentary, and overweight.

These conditions may limit participation and reduce adherence to cardiovascular rehabilitation programs. Unfortunately, the management and evaluation of these musculoskeletal conditions are usually not a priority in these programs [5]. Notoriously, understanding the prevalence of musculoskeletal conditions in cardiac patients undergoing cardiovascular rehabilitation programs is crucial to ensure that interprofessional providers can tailor these programs to meet the needs of these patients.

However, there is no summary information on this subject. Understanding the prevalence of musculoskeletal conditions can contribute to the appropriate selection and application of interventions, including prevention, treatment, and rehabilitation. Despite previous studies addressing this issue, the true prevalence of musculoskeletal conditions among patients in cardiovascular rehabilitation programs remains unclear, as the available evidence is fragmented, heterogeneous, and dated. Therefore, this systematic review aimed to estimate the prevalence of musculoskeletal conditions among patients enrolled in cardiac rehabilitation programs and to evaluate the quality of the available evidence.

## Review

Methods

Protocol Design and Registration

This systematic review follows the recommendation of Meta-Analysis of Observational Studies in Epidemiology (MOOSE) [9]. The systematic review protocol was prospectively registered in the Open Science Framework (OSF; doi: 10.17605/OSF.IO/ZK3BU) on May 30, 2022.

Searches

The search strategy was updated and last executed in May 2024 using a combination of search terms in the following electronic databases: MEDLINE, EMBASE, CINAHL, Web of Science, and SCOPUS, with a focus on musculoskeletal conditions, heart disease, cardiovascular rehabilitation programs, and prevalence. The initial selection of search terms was based on the Medical Subject Headings (MeSH) thesaurus in PubMed. These terms were then adapted to the specific syntax required by each of the other databases searched. Reference lists of included studies and relevant publications on this topic were also screened for any potentially eligible studies. Although no restrictions were applied regarding language or date of publication, all studies meeting the inclusion criteria were published in English.

Eligibility Criteria

The eligibility criteria were applied using the structured search PECO acronym for “population of interest, exposure, comparator, and outcome” [10]. Observational studies that described the prevalence of musculoskeletal conditions in patients with heart disease, aged ≥18 years, of both sexes, submitted to cardiovascular rehabilitation programs regardless of the phase and time of intervention.

Population of Interest

Cardiac patients enrolled in a cardiovascular rehabilitation program were included. Eligible participants were aged 18 years or older and had a confirmed diagnosis of acute coronary syndrome. The sample also included individuals who had undergone percutaneous coronary intervention, coronary artery bypass grafting, or surgery, and those with a history of myocardial infarction. Patients with heart failure related to acute coronary syndrome or with an associated diagnosis of heart failure or arrhythmia were also eligible. In addition, cases of heart transplantation, arterial hypertension, atrial fibrillation, unstable angina, valve repair or replacement surgery, and musculoskeletal conditions were considered [4].

The musculoskeletal conditions [6] are a group of diverse disorders, encompassing inflammatory diseases such as rheumatoid arthritis or gout; age-related conditions such as osteoporosis and osteoarthritis; common conditions of uncertain etiology, such as back pain and fibromyalgia; and those related to activity or injuries, such as occupational musculoskeletal disorders, sports injuries, or the consequences of falls and severe trauma, and are associated with pain, limitations in activities of daily living, and impaired physical function.

Exhibition

Evaluation of musculoskeletal conditions in patients with heart disease enrolled in cardiovascular rehabilitation programs participating in physical exercise during rehabilitation in the following phases: in-hospital, outpatient, and at home with or without telerehabilitation under indirect supervision.

Outcome

In this review, the included studies reported the prevalence of musculoskeletal conditions in patients with heart disease undergoing cardiovascular rehabilitation programs that assessed musculoskeletal aspects such as screening, complaints, functional limitation, impaired physical function, musculoskeletal pain, and age-related conditions such as osteoporosis and osteoarthritis, as well as inflammatory diseases and the affected regions. Both self-reported and clinically diagnosed musculoskeletal conditions were considered eligible for inclusion to capture a comprehensive profile of patients participating in rehabilitation programs.

Study Selection and Data Extraction

Two independent reviewers (ASS, FSP) executed the selection of studies, and in case of disagreement, a third reviewer (ACCGT) was consulted to reach a mutual consensus. The Rayyan Qatar Computing Research Institute (Rayyan QCRI) [11] was the platform used for the option of studies. The two independent reviewers analyzed the titles and abstracts of the articles found in each database, applying the eligibility criteria described above. The complete texts selected in the previous phase were eligible if they met the eligibility criteria described in the protocol. Doubts about eligibility while reading the full text could be clarified by contacting the authors, as the inability to count them would exclude the study.

Two independent reviewers extracted data from the studies using a standardized form, which was divided into two steps for better handling of information. The first step of the form specified the citation details for the name of the author(s), article title, year of publication, magazine/volume/page, and country. The second stage characterized the study regarding the total number of participants in the sample and context, average age group of the total number of participants, research design, the method used to detect musculoskeletal conditions, affected regions, phase, time, intervention and attendance in the programs of cardiovascular rehabilitation, recommendation of the health professional to carry out cardiovascular rehabilitation, search for health services, hospitalization for heart disease and or musculoskeletal conditions, social support inventory, functional status, functional tests, pain assessment and classification scales, medications used, quality of life, glycemia, cholesterol, triglycerides, comorbidities such as chronic respiratory disease, arterial hypertension, diabetes mellitus, chronic kidney disease, depression, dyslipidemia, stroke, neurological disorders, obesity, physical inactivity, smoking. Estimates of the prevalence of musculoskeletal conditions in patients with heart disease undergoing cardiovascular rehabilitation programs were collected, reported, and stratified by gender or age, along with risk ratios and 95% confidence intervals (CIs).

Risk of Bias Assessment

The risk of bias in the observational studies included in this review was assessed using the Newcastle-Ottawa scale [12]. The Newcastle-Ottawa scale was applied by two independent reviewers (ASS, FSP). In case of disagreement, a third reviewer (ACCGT) would be consulted to reach a mutual consensus. The Newcastle-Ottawa scale was used to assess each study in three domains: selection, comparability, and outcome (for cohort studies) and exposure (for case-control studies). The Newcastle-Ottawa scale uses a star system to allow a semi-quantitative assessment of study quality, which ranges from zero to nine stars. Higher scores represent better quality. Articles that achieved a score of seven or more stars were considered articles of excellent methodological quality. Articles with a score of five to six stars were considered of good methodological quality.

Analysis of Results

All meta-analyses were conducted using the RStudio software version 1.2.5042 (Posit, PBC, MA, USA). Pooled estimates were calculated using a random effect model, using the I² statistic test. Due to the heterogeneity of the studies (represented by I²), they can be classified as homogeneous when I²=0%, low heterogeneity when 1% to 25%, moderate heterogeneity when 26% to 49%, and high heterogeneity when I² >50% [13-16].

The prevalence of musculoskeletal conditions allowed the grouping of data in meta-analysis under a general perspective and complementary analyses. This took into account the affected region and the type of pathology, respecting the classification of the primary studies included. Thus, they were represented by the percentage and their respective 95% CI. For studies that had different outcomes and could not be grouped in the meta-analysis chart, we present the individual data of the studies in tables.

Evidence Quality Analysis

The quality of the evidence was graded according to the Grading of Recommendations Assessment, Developing and Evaluation system (GRADE) as high (4 points - further research unlikely to change confidence in the evidence), moderate (3 points - further research is likely to have a significant impact on changing confidence in the evidence), low (2 points - very likely that further research will have a significant impact on changing confidence in the evidence), or very low (1 point - the confidence that can be used in the evidence is uncertain) [17]. Whereas the GRADE system was developed to assess the quality of evidence from systematic intervention reviews, previous reviews that included observational studies were used as a parameter to assess the quality of evidence [18-21]. Therefore, the following criteria were used to assess the domains and determine the quality of evidence: risk of bias (when more than 25% of participants are from studies judged to be at high risk of bias), imprecision (when more than 25% of participants are from studies with less than 163 participants), inconsistency (i.e., substantial heterogeneity, I² > 50%), indirect evidence (i.e., study population and outcome measures aligned with the purpose of the review), and publication bias (i.e., evidence of publication bias by funnel plot asymmetry).

Results

**Figure 1:**
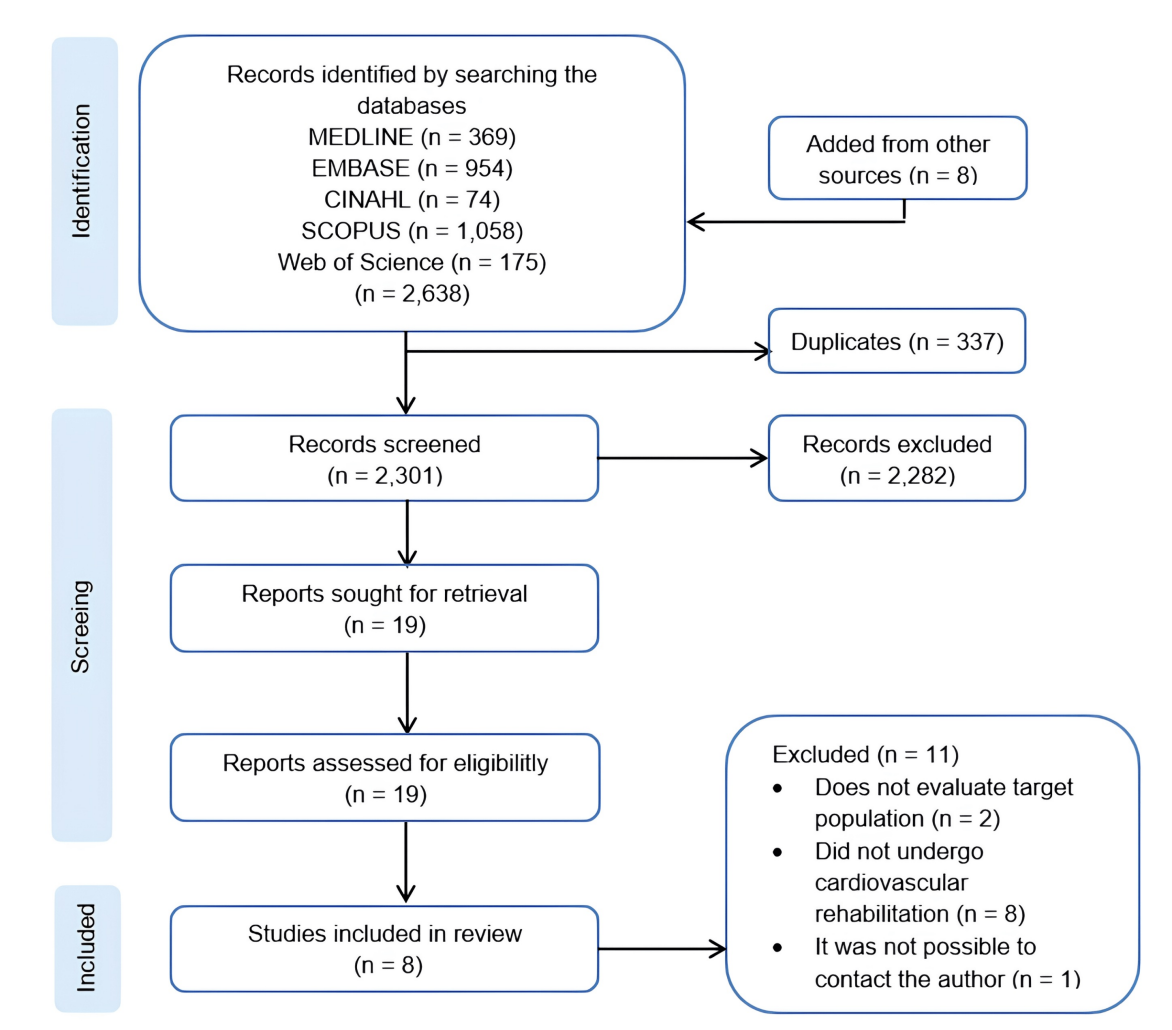
PRISMA study flow diagram PRISMA: Preferred Reporting Items for Systematic Reviews and Meta-Analyses

The search in electronic databases yielded 2,630 records. After reviewing the bibliographic references, eight articles were added, bringing the total to 2,638 studies for selection. We identified 337 duplicate records. After screening titles and abstracts, we identified 19 potentially eligible articles for text review. We excluded 11 articles because they did not assess the target population (n=2), did not include individuals who underwent cardiovascular rehabilitation (n=8), or made it impossible to contact the author (n=1). Finally, we included 8 [22-27] studies for qualitative analysis and 7 [5,7,23-27] studies for quantitative analysis, as shown in Figure 1.

Three studies [5,7,22] were longitudinal observational prospective cohort studies, two were longitudinal observational retrospective cohort studies [26-27], and three studies were cross-sectional observational [23-25]. Table 1 provides an overview of the included studies.

**Table 1:** Characterization of included studies MSKC: musculoskeletal condition, SD: standard deviation, BMI: body mass index, CR: cardiovascular rehabilitation, NA: not applicable

Study name	Country - source	Study design	Collection tool MSKC	Sample characteristic	MSKC in CR	Prevalence (% ( n ))	
Supervia et al. [5], 2021	United States, Minnesota, Rochester	Longitudinal observational study, prospective cohort	Application of questionnaires	Full sample: 321; mean age (SD): 68 (10.8); men: 228 (71%); women: 93 (29%); mean BMI (SD): 29.1 (5.2); myocardial infarction/revascularization of the myocardium/percutaneous coronary intervention; time (CR): NA; frequency (CR): NA; type of exercise (CR): NA; exercise time (CR): NA	Pain on performing moderate exercise; arthritis; balance problems; disc herniation; swelling in the joints; inflammatory arthritis; osteoporosis; spinal stenosis; vertebral fracture; neurological problem (except stroke)	60.0 (193); 45.2 (145); 37.0 (119); 14.0 (45); 11.5 (37); 7.5 (24); 8.7 (28); 6.2 (20); 3.4 (11); 1.9 (6)	
Rocha et al. [25], 2015	Portugal, Porto	Cross-sectional observational study	Application of questionnaires	Full sample: 449; mean age (SD): 54.1 (9.7); men: 395 (88%); women: 54 (12%); mean BMI (SD): 27.1 (3.8); myocardial infarction/revascularization of the myocardium/percutaneous coronary intervention/arterial hypertension; time (CR): NA; frequency (CR): NA; type of exercise (CR): NA; exercise time (CR): NA	Lower limb musculoskeletal pain; knee pain; ankle/foot pain; back pain; upper limb musculoskeletal pain	58.0 (68); 68.7 (46); 20.3 (14); 34.0 (40); 7.7 (9)	
Wittkopf et al. [24], 2018	United Kingdom, Leeds, Brazil, Florianopolis	Cross-sectional observational study	Application of questionnaires	Full sample: 105; mean age (SD): 67.89 (9.68); men: 78 (74.3%); women: 27 (25.7%); mean BMI (SD): 28.56 (4.83); coronary artery disease/revascularization of the myocardium/percutaneous coronary intervention/bypass graft/arterial hypertension; time (CR): NA; frequency (CR): NA; type of exercise (CR): NA; exercise time (CR): NA	Osteoarthritis; osteoporosis; strains/sprains; gout; lumbar disc herniation; rheumatoid arthritis	45.45 (21); 13.6 (6); 13.6 (6); 9.1 (4); 9.1 (4); 9.1 (4)	
Marzolini et al. [23], 2012	Canada, Ontario, Windsor, Sudbury, Ottawa	Cross-sectional observational study	Self-reports	Full sample: 1803; mean age (SD): 65.4 (10.4); men: 1355 (75.15%); women: 448 (24.8%); mean BMI (SD): 28.2 (5.3); acute coronary syndrome/unstable angina/arrhythmia/heart valve repair/revascularization of the myocardium/percutaneous coronary intervention/cardiac insufficiency; time (CR): NA; frequency (CR): NA; type of exercise (CR): NA; exercise time (CR): NA	Arthritis/joint pain; spine disorders; not specified	64.4 (650); 6.4 (65); 21.9 (221)	
Goel et al. [26], 2010	United States, Minnesota, Rochester	Longitudinal observational study, retrospective cohort	Medical Records	Full sample: 284; mean age (SD): 62.1 (12.3); men: 205 (72%); women: 79 (28%); mean BMI (SD): NA; myocardial infarction/revascularization of the myocardium / percutaneous coronary intervention/heart transplant; time (CR): NA; frequency (CR): NA; type of exercise (CR): NA; exercise time (CR): NA	Balance problems; lumbar region pain; knee pain; hip pain; shoulder pain; foot pain; ankle pain; thigh pain; hand pain; neck pain; pain in other locations (chest, arm, elbow, wrist); vertebral fractures; limb amputations; osteoporosis; peripheral neuropathy; stroke/weakness of extremities; stroke/sensory deficits	58.0 (164); 29.0 (20); 17.0 (12); 7.7 (5.39); 7.0 (4.9); 4.9 (3.43); 2.5 (1.75); 2.8 (1.96); 2.5 (1.75); 2.0 (1.4); 3.9 (2.73); 7.0 (4.9); 2.0 (1.4); 11.0 (7.7); 5.0 (14); 4.0 (1); 3.0 (8.5)	
Khan et al. [27], 2014	Canada, Toronto	Longitudinal observational study, retrospective cohort	Medical records	Full sample: 51; mean age (SD): 62.9 (11); men: 33 (64.7%); women: 18 (35.3%); mean BMI (SD): 28.91 (6.8); congestive heart failure/coronary artery disease/revascularization of the myocardium/percutaneous coronary intervention/bypass graft/arterial hypertension/valve surgery/heart transplant; time (CR): 1 year; frequency (CR): weekly, monthly classes and independent management; type of exercise (CR): aerobic and resistance exercises; exercise time (CR): 90 minutes, once a week	Knee osteoarthritis; meniscal injuries; spinal stenosis; spinal osteoarthritis; shoulder adhesive capsulitis; rotator cuff tendonitis; backache; hip pain; knee pain; feet pain; thigh pain; leg pain; pain in buttocks; ankle pain; shoulder pain; upper limb pain; neck pain	27.45 (14); 7.84 (4); 9.8 (5); 5.38 (3); 11.76 (6); 11.76 (6); 25.5 (13); 9.8 (5); 45.1 (23); 11.8 (6); 5.9 (3); 13.7 (7); 13.7 (7); 9.8 (5); 17.6 (9); 9.8 (5); 3.9 (2)	
Marzolini et al. [7], 2010	Canada, Toronto	Longitudinal observational study, prospective cohort	Application of questionnaires	Full sample: 322; mean age (SD): 63.4 (12); men: 233 (72.4%); women: 89 (27.6%); mean BMI (SD): 29.2 (6.9); coronary artery disease/myocardial infarction/percutaneous coronary intervention/revascularization of the myocardium/aortic valve surgery/atrial fibrillation; time (CR): 6 months; frequency (CR): weekly classes; type of exercise (CR): aerobic and resistance exercises; exercise time (CR): 30 to 60 minutes	Arthritis; strains/sprains; location MSKC: knee, low back, hip, shoulder, legs, feet, ankles, hands, arms, neck, calf, chest; characteristic MSKC: single MSKC, two MSKC, three MSKC, upper body, lower body, upper and lower body, unilateral, bilateral, unilateral and bilateral	36.6 (59); 28.6 (46); 25.0 (59); 19.0 (45); 12.3 (29); 11.0 (26); 8.5 (20); 5.5 (13); 5.0 (12); 4.7 (11); 2.1 (5); 2.1 (5); 1.7 (4); 1.7 (4); 60.9 (98); 26.7 (43); 12.4 (20); 16.1 (26); 70.2 (113); 13.0 (21); 60.9 (98); 32.9 (53); 5.6 (9)	
Marzolini et al. [22], 2013	Canada, Toronto	Longitudinal observational study, prospective cohort	Self-reports	Full sample: 1680; participants with MSKC full sample: 851; mean age (SD): 66.4 (10.3); men: 579 (68%); women: 272 (32.2%); mean BMI (SD): 28.6 (5.6); participants CR with MSKC full sample: 424 (49.8%); mean age (SD): 64.8 (9.7); men: 301 (70.9%); women: 123 (29.1%); mean BMI (SD): 28.6 (5.4); participants CR without MSKC full sample: 427 (50.2%); acute coronary syndrome/percutaneous coronary intervention/revascularization of the myocardium / cardiac insufficiency/arrhythmia / arterial hypertension/valve repair/replacement; time (CR): NA; frequency (CR): NA; type of exercise (CR): NA; exercise time (CR): NA	Arthritis and joint pain	68.0 (281)	

The eight [5,7,22-27] eligible studies were published between 2010 and 2021. The studies are from the United States (Rochester/Minnesota [5,26]), from Canada (Toronto [7,22,27], Ontario/Windsor/Sudbury, and Ottawa [23]), the United Kingdom (Leeds [24]), and Brazil (Florianópolis [24]). Study participants had heart disease and musculoskeletal conditions and belonged to a cardiovascular rehabilitation program. These studies comprised a total of 3,759 participants, 2,828 men and 931 women, aged between 54 and 68 years old, with body mass indexes from 27.1 to 29.2 kg/m².

The included studies reported the prevalence of musculoskeletal comorbidities [7,22], musculoskeletal conditions [23,27], musculoskeletal limitations [5], musculoskeletal complaints [25], chronic musculoskeletal pain [24], musculoskeletal disorders [26], and balance [5,26] in cardiac patients submitted to cardiac rehabilitation programs. Five studies [5,24-27] described data from people with musculoskeletal pain, four studies reported knee pain [7,25-27], and five studies [7,23,25-27] reported on back pain. Four studies used scales to assess pain intensity [5,25-27] in patients enrolled in a cardiac rehabilitation program. Four studies [5,7,22-23] reported patients with arthritis. Two studies [24,27] showed osteoarthritis data of participants. Three studies [5,24,26] expressed data from people with osteoporosis. A study [5] identified musculoskeletal limitations using a seven-item screening tool. Two studies [7,27] evaluated the maximum oxygen consumption at baseline and after the intervention of patients enrolled in cardiac rehabilitation with musculoskeletal conditions. Methods used to assess musculoskeletal conditions included medical records [26-27], self-reports [22-23], and application of questionnaires [5,7,24-25]. The studies reported the following cardiovascular diseases: myocardial infarction [5,7,25-26], arterial hypertension [24-25,27], coronary artery disease [7,24,27], acute coronary syndrome [22-23], unstable angina [23], arrhythmia [22-23], atrial fibrillation [7], cardiac insufficiency [22-23], and congestive heart failure [27]. Surgical cardiac procedures reported in the studies were myocardial revascularization [7,22-27], percutaneous coronary intervention [7,22-27], bypass graft [24,27], heart transplant [26-27], and valve surgery [7,22-23,27]. As for the risk of bias analysis, using the Newcastle-Ottawa scale, represented by Table 2.

**Table 2:** Risk of bias analysis for observational studies using the Newcastle-Ottawa scale Excellent methodological quality: seven or more stars; good methodological quality: five to six stars; and low methodological quality: four stars or less [12].

Reference	Design	Selection	Comparability	Outcome	Total
Supervia et al. [5], 2021	Prospective cohort	***		*	4
Rocha et al. [25], 2015	Cross-sectional	****	*	**	7
Wittkopf et al. [24], 2018	Cross-sectional	****	* *	**	8
Marzolini et al. [23], 2012	Cross-sectional	***	*	**	6
Goel et al. [26], 2010	Retrospective cohort	****		**	6
Khan et al. [27], 2014	Retrospective cohort	***		**	5
Marzolini et al. [7],2010	Prospective cohort	****	**	**	8
Marzolini et al. [22], 2013	Prospective cohort	****	**	***	9

It was possible to classify 50% of the included studies [7,22,24-25] as having excellent methodological quality, 37.5% [23,26-27] as good quality, and 12.5% as low quality [5]. Among the aspects analyzed and not covered by the selected studies, we found the absence of a control group or a non-exposed group for the comparability item. Only one study lacked a statement about the loss of follow-up of study participants, and two studies indicate greater subjectivity in the collection of prevalence data because they used self-report as a method for collecting data on the main outcome.

**Figure 2:**
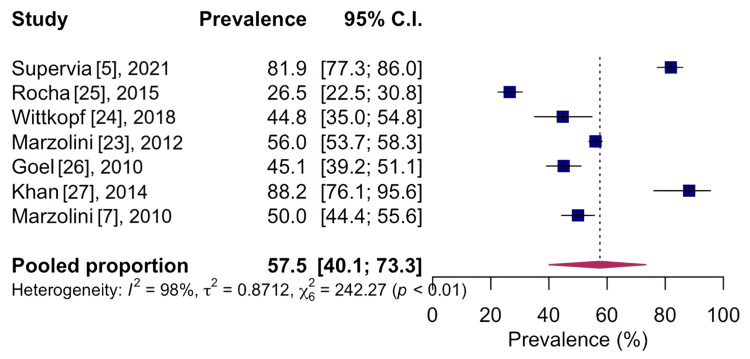
Forest chart of the prevalence of general musculoskeletal conditions CI: confidence interval

In the quantitative analysis of the included studies, it was possible to find the prevalence of 57.5%, CI 95%: 40.1-73.3; I²: 98%, based on seven studies with n=3,335, indicating low quality of evidence, for the analyzed musculoskeletal conditions on general aspects, as shown in Figure 2.

Complementary meta-analyses have been performed about the affected regions and pathologies of the musculoskeletal conditions reported by the selected studies. For musculoskeletal conditions, it was possible to summarize the knee and spine regions. In this context, data on musculoskeletal pain, arthritis, and osteoporosis were still analyzed in a meta-analysis regarding the types of pathologies.

**Figure 3:**
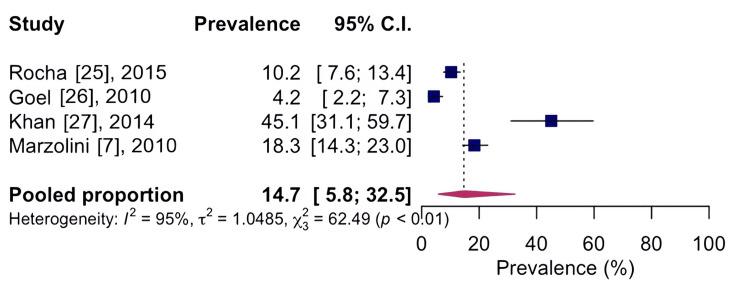
Forest chart of the prevalence of musculoskeletal conditions for the knee region CI: confidence interval

**Figure 4:**
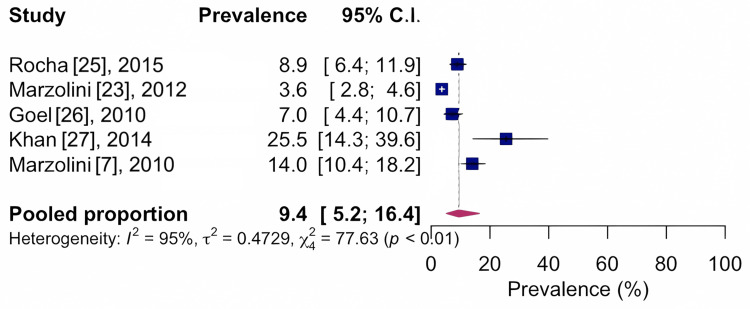
Forest chart of the prevalence of musculoskeletal conditions for the spine region CI: confidence interval

Regarding the affected regions mentioned by the included studies, the prevalence was found to be 14.7%, CI 95%: 5.8-32.5; I²: 95%, based on four studies with n=1,106, indicating low quality of evidence, for the knee area, as shown in Figure 3, and 9.4%, CI 95%: 5.2-16.4; I²: 95%, based on five studies with n=2,909, indicating low quality of evidence, for the spine, as shown in Figure 4.

**Figure 5:**
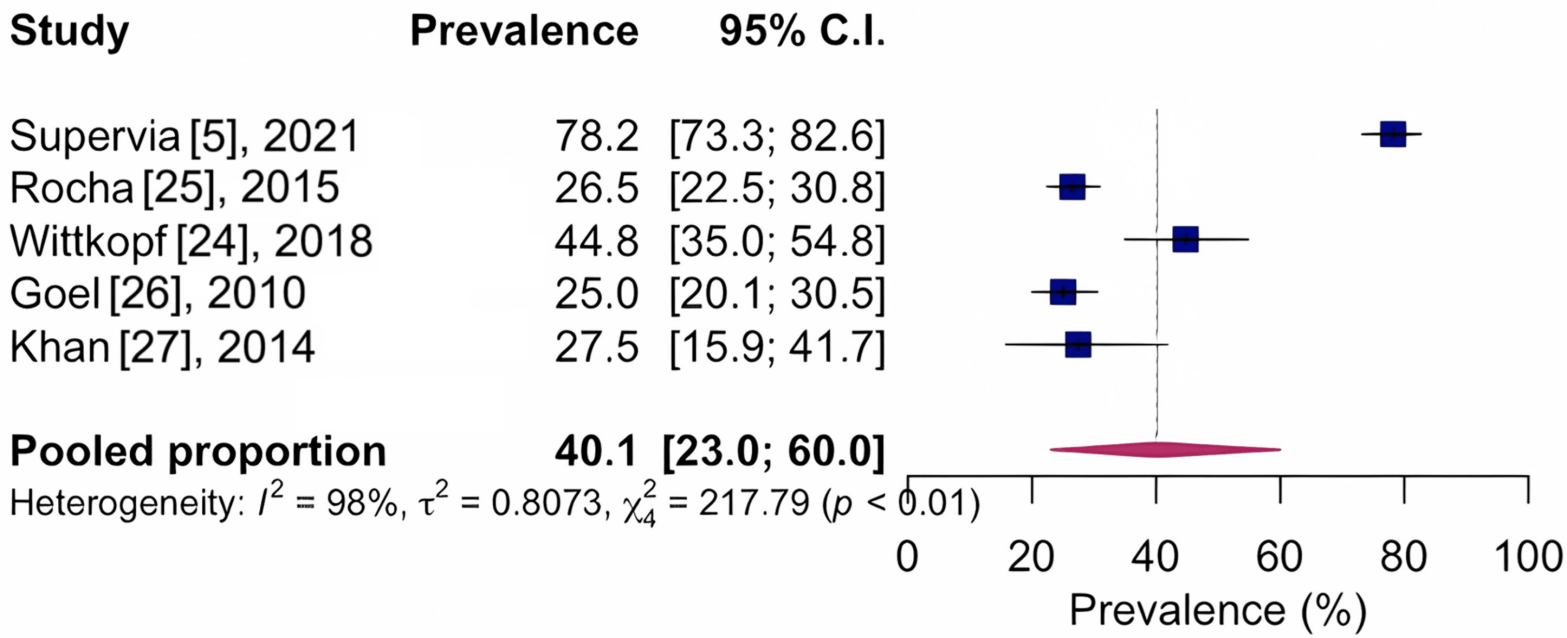
Forest chart of prevalence of musculoskeletal conditions for musculoskeletal pain pathology CI: confidence interval

Regarding the reported pathologies, the prevalences were compiled as 40.1%, CI 95%: 23.0-60.0; I²: 98%, based on five studies with n=1,210, indicating very low quality of evidence for musculoskeletal pain, as shown in Figure 5.

**Figure 6:**
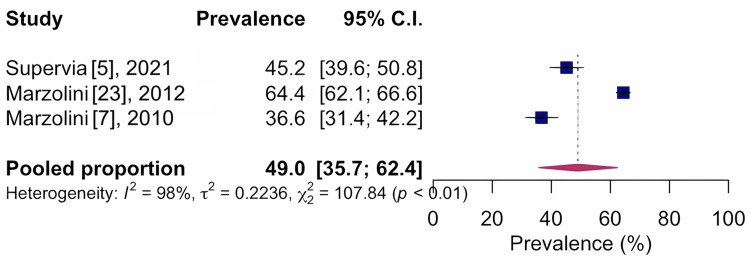
Forest chart of prevalence of musculoskeletal conditions for musculoskeletal pain pathology CI: confidence interval

**Figure 7:**
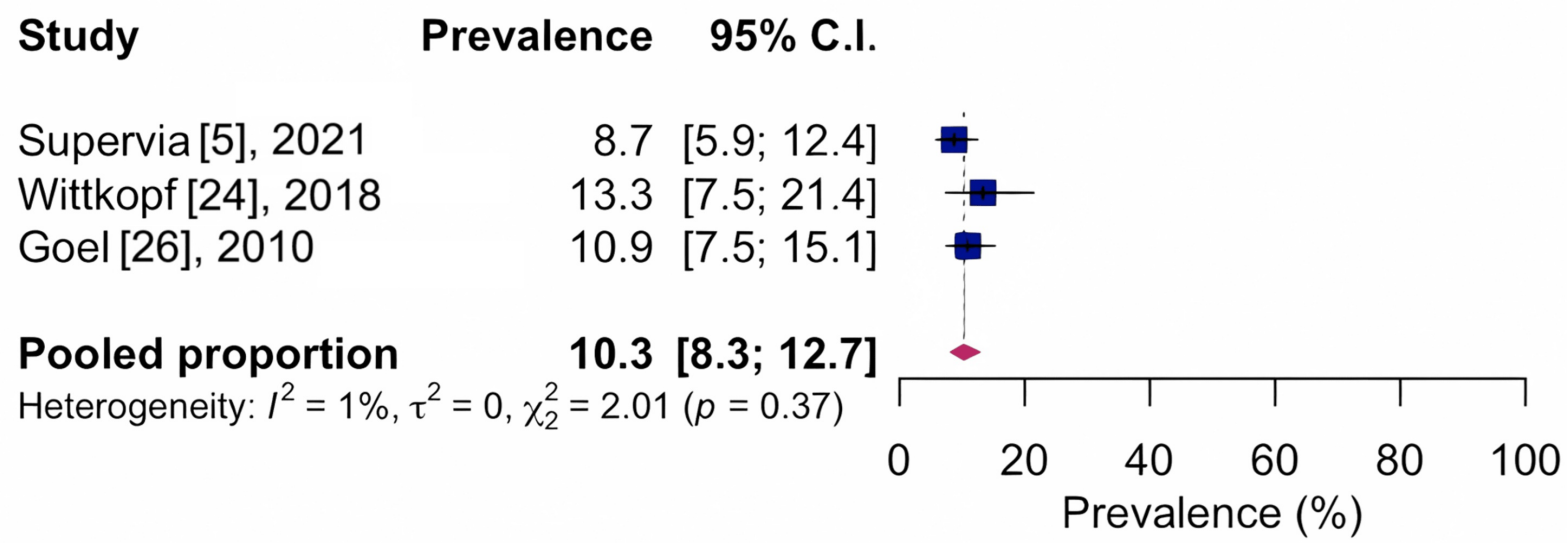
Forest chart of prevalence of musculoskeletal conditions for musculoskeletal pain pathology CI: confidence interval

For arthritis, 49.0%, CI 95%: 35.7-62.4; I²: 98%, based on three studies with n=2,446, indicates very low quality of evidence, as shown in Figure 6, and 10.3%, CI 95%: 8.3-12.7; I²: 1%, based on three studies with n=710, indicating moderate quality of evidence for osteoporosis, as shown in Figure 7.

The included studies showed heterogeneity in the study designs and instruments for assessing musculoskeletal conditions. For this reason, not allowing musculoskeletal conditions could be represented in a meta-analysis. Furthermore, the study by Marzolini et al. [22] was not included in the quantitative analysis because the presented prevalences were generated by conveniently dividing the sample into two groups: those with musculoskeletal conditions and those without. Only the outcomes of mortality and quality of life were presented for the general sample. In studies with varying results, the data were presented individually in tables.

The pooled analyses revealed consistently high heterogeneity for several outcomes (I² = 95-98%), likely reflecting differences in study designs (prospective, retrospective, and cross-sectional), variations in participant age and baseline characteristics, and the use of diverse tools to assess musculoskeletal conditions (medical records, self-reports, and questionnaires). These methodological and population differences likely contributed to the observed variability, and therefore, prevalence estimates should be interpreted with caution in the context of the included studies. Despite this heterogeneity, combining observational data provides a meaningful overall estimate of musculoskeletal condition prevalence in patients undergoing cardiovascular rehabilitation.

According to the GRADE assessment, the overall quality of evidence for the primary and secondary outcomes ranged from very low to moderate, mainly due to inconsistency across the meta-analyses in Table 3.

**Table 3:** Assessment of the quality of evidence using GRADE for primary and secondary outcomes ^a ^The summary shows 98% heterogeneity, indicating inconsistency of information and resulting in a two-level downgrade of the evidence. ^b ^The summary shows 95% heterogeneity, indicating inconsistency of information and resulting in a two-level downgrade of the evidence. ^c ^The summary shows 96% heterogeneity, indicating inconsistency of information and resulting in a two-level downgrade of the evidence. ^d ^The summary presents a study of low methodological quality, resulting in a one-level downgrade of the evidence. ^e ^The summary shows 98% heterogeneity, indicating inconsistency of information and resulting in a two-level downgrade of the evidence.

№ of studies	Certainty assessment								Effect			Certainty	Importance
	Study design	Risk of bias	Inconsistency		Indirect evidence		Imprecision	Other considerations	Prevalence (%)	Total	CI 95%		
Prevalence of general musculoskeletal conditions													
7	Observational study	Not serious	Very serious^a^		Not serious		Not serious	None	57.5	3,335	40.1-73.3	⨁⨁◯◯ low	Important
Prevalence of musculoskeletal conditions in the knee													
4	Observational study	Not serious	Very serious^b^		Not serious		Not serious	None	14.7	1,106	5.8-32.5	⨁⨁◯◯ low	Important
Prevalence of musculoskeletal conditions in the spine													
5	Observational study	Not serious	Very serious^c^		Not serious		Not serious	None	9.4	2,909	5.2-16.4	⨁⨁◯◯ low	Important
Prevalence of musculoskeletal conditions in relation to musculoskeletal pain													
5	Observational study	Serious^d^		Very serious^c^	Not serious	Not serious		None	40.1	1,210	23.0-60.0	⨁◯◯◯ very low	Important
Prevalence of musculoskeletal conditions in relation to arthritis													
3	Observational study	Serious^d^	Very serious^e^		Not serious		Not serious	None	49.0	2,446	35.7-62.4	⨁◯◯◯ very low	Important
Prevalence of musculoskeletal conditions in relation to osteoporosis													
3	Observational study	Serious^d^	Not serious		Not serious		Not serious	None	10.3	710	8.3-12.7	⨁⨁⨁◯ moderate	Important

Formal assessment of publication bias was not feasible given the limited number of studies (<10), in line with Cochrane recommendations.

Discussion

According to our knowledge and research, this is the first systematic review study on the prevalence of musculoskeletal conditions for patients enrolled in a cardiovascular rehabilitation program. Among the eight articles included in this review, differences were found in the type of study designs (longitudinal observational prospective cohort, longitudinal observational retrospective cohort, cross-sectional observational), and variations in the definitions of musculoskeletal conditions contributed to the high heterogeneity observed across studies. A total of 24 musculoskeletal conditions have been reported, with arthritis, osteoporosis, and musculoskeletal pain being the most frequently mentioned. And the regions commonly affected were the spine and knee. Old age and a high body mass index [23] foreshadowed musculoskeletal conditions. In general, these studies have a low risk of bias.

Musculoskeletal conditions are common in patients enrolled in a cardiovascular rehabilitation program [23]. Previous studies estimated that approximately half of these patients reported a musculoskeletal condition [7]. The data pooled in our systematic review showed a prevalence of 57.5% of musculoskeletal conditions in individuals undergoing a cardiovascular rehabilitation program. High heterogeneity may be related to differences in study designs, age groups, and musculoskeletal condition collection tools.

Different data collection methods (medical records, self-report, questionnaires) may introduce information bias and misclassification, reducing comparability. Information bias [28] results from systematic differences in how data from studies were collected or from measurement errors. Data extracted from medical records depends on individual provider reports and may not have been reported consistently. Measurements from collection instruments depend on the accuracy of participants' responses about musculoskeletal conditions. Thus, the different tools for collecting musculoskeletal conditions used in the included studies can lead to low accuracy and sensitivity, introduce information bias and misclassifications, and possibly justify the low quality of evidence on general aspects of musculoskeletal conditions.

The most common locations of musculoskeletal conditions were the knee region, with a prevalence of 14.7%, and the spine, with a prevalence of 9.4%. Musculoskeletal conditions, especially arthritis, are common in individuals with coronary artery disease [7,23]. Our results showed that 49.0% of all cardiovascular rehabilitation program participants reported arthritis [29]. Arthritis is considered one of the principal causes of disability. In this review, we observed that patients enrolled in cardiovascular rehabilitation programs were generally overweight, elderly, and had musculoskeletal problems. Being overweight can be a contributing factor to the generation of chronic joint overload and possible risk for musculoskeletal conditions. As for the age range, the population included was characterized as senile. In the senile population, musculoskeletal conditions [29] are among the most common problems, including arthritis.

In our systematic review, the prevalence of musculoskeletal pain in patients enrolled in cardiovascular rehabilitation programs was relatively high, at 40.1%. There are studies [30] that have described the co-occurrence of chronic musculoskeletal pain and cardiovascular diseases and have reported high-quality evidence that people with chronic musculoskeletal pain are 1.91 times more likely to develop a cardiovascular disease. Thus, it may be one of the possible justifications for the high prevalence of musculoskeletal pain in participants of a cardiovascular rehabilitation program.

According to the summary of studies, another frequently reported musculoskeletal condition is osteoporosis. The prevalence of osteoporosis was 10.3% in individuals undergoing the cardiovascular rehabilitation program, with low heterogeneity (I² = 1%) and moderate quality of evidence. Osteoporosis [31] is prevalent in aging, with a worldwide prevalence of 21.7%. The absence of a control group did not affect confidence in the effect estimate, as representativeness was ensured by the participant selection method, with no significant sample losses. Thus, it is possible to understand that the prevalence of musculoskeletal conditions found in meta-analyses is reliable and reflects the demands and needs of patients included in a cardiovascular rehabilitation program.

The high prevalence of musculoskeletal conditions in cardiac patients undergoing cardiovascular rehabilitation programs is a notable finding for interprofessional providers, for training, and for the adequacy of assessments and treatments proposed for this population. Our findings suggest that musculoskeletal conditions may be related to the underutilization of cardiovascular rehabilitation programs. However, future studies are needed to explore musculoskeletal conditions as possible barriers to adherence to these programs.

Another factor to be considered is the infrequent use of instruments for assessing and measuring musculoskeletal conditions in patients enrolled in cardiovascular rehabilitation programs. In this sense, it becomes relevant to use specific assessment tools to obtain detailed information about the presence, severity, and symptoms of musculoskeletal conditions and their interference with the functional aspects of this population included in a cardiovascular rehabilitation program.

The high prevalence of musculoskeletal comorbidities in patients undergoing cardiovascular rehabilitation programs has important clinical implications. These conditions can limit exercise capacity, affect tolerance to different training modalities, and require individualized monitoring and adaptations to ensure patient safety and optimize outcomes. Clinicians should consider baseline musculoskeletal assessments when designing rehabilitation programs, adjusting intensity, frequency, and type of exercises according to patients’ functional limitations, and providing appropriate supervision. Furthermore, musculoskeletal conditions may act as barriers to program adherence, highlighting the need for integrated care strategies and interdisciplinary collaboration.

Study limitations

The studies included in this systematic review presented several limitations, such as differences in study designs, age groups, data collection tools, and a lack of standardization in the nomenclature of musculoskeletal conditions. These factors may have contributed to the observed heterogeneity of results. Moreover, the scarcity of detailed data on exercise prescription, such as type, intensity, duration, and frequency, limited the ability to explore potential relationships between prescribed exercise, musculoskeletal condition exacerbations, and the adequacy of treatment for heart disease. Potential biases within the included studies may also have influenced the findings. The use of heterogeneous data collection methods, ranging from medical records to self-reports and questionnaires, introduces measurement bias and may affect the accuracy of prevalence estimates. Furthermore, the absence of control groups in most studies restricts comparative analyses, while variability in study designs and populations further contributes to heterogeneity. Despite these limitations, the findings provide valuable insights for clinical practice and highlight the importance of adopting standardized assessment protocols and more rigorous research designs in future investigations.

## Conclusions

This systematic review and meta-analysis found a high prevalence of musculoskeletal conditions, particularly arthritis, musculoskeletal pain, and osteoporosis, among patients enrolled in cardiac rehabilitation programs. The most affected regions were the knee and spine. These findings highlight the importance of musculoskeletal assessment and individualized exercise prescription within cardiac rehabilitation. Future studies should clarify whether exercise type, frequency, and intensity influence musculoskeletal outcomes in this population.
